# Phenotypes of peripheral CD4^+^ T helper cell subsets in pregnant women with HBeAg-negative chronic asymptomatic HBV carriers

**DOI:** 10.3389/fcimb.2023.1126311

**Published:** 2023-02-01

**Authors:** Guofang Feng, Yu Sun, Shifen Wang, Yan Lv, Cuilin Yan, Yimin Zhu, Yongsheng Zheng, Dawei Cui

**Affiliations:** ^1^ Department of Reproductive Endocrinology, Women’s Hospital, Zhejiang University School of Medicine, Hangzhou, China; ^2^ Department of Blood Transfusion, The First Affiliated Hospital, Zhejiang University School of Medicine, Hangzhou, China; ^3^ Key Laboratory of Clinical In Vitro Diagnostic Techniques of Zhejiang Province, Hangzhou, China; ^4^ Department of Laboratory Medicine, The First Affiliated Hospital, Zhejiang University School of Medicine, Hangzhou, China; ^5^ Department of Clinical Laboratory, The Affiliated Suzhou Hospital of Nanjing Medical University, Suzhou Municipal Hospital, Gusu School, Nanjing Medical University, Suzhou, Jiangsu, China

**Keywords:** hepatitis B virus, pregnancy, asymptomatic HBV carriers, CD4 + T cells, cytokines

## Abstract

**Background:**

Chronic hepatitis B virus (HBV) infection is a major public health problem worldwide, and mother-to-child transmission is the key mode of HBV infection. CD4^+^ T helper (Th) cells play a critical role in the immune microenvironment of specific maternal tolerance to the foetus during pregnancy. However, the roles of Th cell subsets in pregnant women (PW) with chronic asymptomatic HBV carriers (ASCs) remain completely unclear. Here, we aimed to characterize CD4^+^ T-cell immunity in PW with hepatitis Be antigen (HBeAg)-negative chronic ASCs.

**Methods:**

Human peripheral blood mononuclear cells (PBMCs) from PW without HBV infection or with chronic ASCs and healthy controls (HC) were isolated, and CD4^+^ Th cell subsets were detected by flow cytometry in addition to serum cytokines. Serological HBV markers, liver function and hormone levels of these individuals were also tested.

**Results:**

The frequencies of circulating T follicular helper (Tfh) type 2 (Tfh2) cells were significantly evaluated, but Tfh1 cell frequencies were notably decreased in PW compared to HC. Moreover, the frequencies of Th22 cells were only notably increased in PW with chronic ASCs in comparison with PW. Additionally, increased levels of serum IL-4 were positively correlated with Tfh2 cell frequencies in healthy PW. Interestingly, serum P4 levels were positively associated with the frequencies of circulating Tfh2 or Th2 cells but were negatively related to the frequencies of circulating Tfh17 or Th17 cells in healthy PW. Although there were some changes in the other CD4^+^ Th cell frequencies and cytokine levels or other references, significant differences were not found among HC, healthy PW, PW with HBeAg-negative chronic ASCs.

**Conclusion:**

CD4^+^ Th cell subsets played a critical role in the immune microenvironment of PW, and these findings provided potential evidence for why PW with chronic ASCs did not receive antenatal antiviral prophylaxis.

## 1 Introduction

Hepatitis B virus (HBV) infection remains a major public health issue, causing high morbidity in approximately one-third of the global population worldwide (Yuen et al., 2018; Alaama et al., 2022; Iannacone and Guidotti, 2022). In 2016, the World Health Organization (WHO) launched the global health sector strategy to eliminate viral hepatitis by 2030, with a target of 90% reduction of the incidence of new cases of chronic HBV infection and 65% reduction of HBV infection mortality and a target of 0.1% prevalence of hepatitis B surface antigen (HBsAg) among children in comparison with the 2015 baseline (Organization, 2016). The population with HBV infection is prone to develop chronic infection, and asymptomatic HBV carriers (ASCs) without hepatitis Be antigen (HBeAg) are the majority of chronic HBV-infected individuals, including pregnant women (PW) (Tang et al., 2018; Nguyen et al., 2020; Koffas et al., 2021; Liu et al., 2021; Cui et al., 2022). Moreover, 95% of neonates with HBV infection by mother-to-child transmission (MTCT), which is the predominant mode of HBV infection, are at high risk of developing chronic infection, and they are associated with an increased risk of HBV-related fatal liver diseases, including liver cirrhosis and hepatocellular carcinoma (Organization, 2016; Nguyen et al., 2020; Terrault et al., 2021). Therefore, focus on PW with chronic HBV infection is crucial for the prevention of MTCT of HBV, which is also one key strategy of global HBV elimination by 2030 (Organization, 2016; Organization WH, 2020; Funk et al., 2021). Previous reports showed that in general, chronic HBeAg-negative ASCs had normal alanine aminotransferase (ALT) levels and low HBV-DNA copies and were not treated with antiviral drugs, but follow-up for these patients, including PW without prenatal antiviral prophylaxis, is necessary (Cui et al., 2016; Terrault et al., 2021). The immune mechanism of PW without prenatal antiviral prophylaxis remains unclear.

Accumulating evidence shows that CD4^+^ Th cell subsets, such as Th1, Th2, Th17, T regulatory cells (Tregs) and T follicular helper (Tfh) cells, and their associated cytokines play crucial roles during pregnancy and contribute to developing a tolerant immunological microenvironment towards the foetus (Monteiro et al., 2017; Wang et al., 2020; Monteiro et al., 2021). Chronic HBV infection during pregnancy is an important agent for the number and function of CD4^+^ Th cells that can compromise the safety of the foetus by affecting the immunological environment tolerated by the PW and the foetus (Shrivastava et al., 2013; Liang et al., 2020; Huang et al., 2021). However, the role of CD4^+^ Th cell subsets remains completely unclear in PW with HBeAg-negative chronic ASCs. Therefore, CD4^+^ Th cell subsets should be further analysed in PW who are chronic ASCs and HBeAg-negative, which will contribute to the safety of the foetus by developing a tolerant immunological environment for the PW and the foetus.

In this study, the frequencies of CD4^+^ Th cell subsets were analysed in peripheral blood mononuclear cells (PBMCs) from PW who were HBeAg-negative chronic ASCs, and CD4^+^ Th cell subset-associated cytokines were also tested, which could provide a potential immune explanation for why PW with chronic ASCs did not receive antenatal antiviral prophylaxis, which is the conundrum of foetal tolerance during pregnancy with chronic HBV infection.

## 2 Materials and methods

### 2.1 Demographic data

Twenty-one PW who were HBeAg-negative chronic ASCs with normal ALT levels were enrolled according to the guidelines for the prevention and treatment of chronic hepatitis B (2019 version) (Cui et al., 2022), and fifteen sex- and age-matched healthy PW without HBV infection and 13 healthy controls (HC) without pregnancy were also recruited at Women’s Hospital, Zhejiang University School of Medicine (Hangzhou, China) in this study. Individuals were excluded from the study if they had haematological system diseases, infectious diseases, diabetes, autoimmune disease, or other hepatotropic diseases. Chronic HBeAg-negative ASCs with normal ALT levels were also excluded if they accepted HBV treatment within six months before blood sampling, and healthy PW and HC who had a vaccine history within six months were also excluded. Serum levels of 17-β-oestradiol (E2) and progesterone (P4) were detected by chemiluminescent microparticle immunoassay (Abbott i4000SR, USA). The demographic and laboratory characteristics of PW with chronic ASCs, healthy PW and HC are shown in **Table 1**. This study was approved by the Medical Ethical Committee of the Women’s Hospital, Zhejiang University School of Medicine according to the Declaration of Helsinki (1964).

**Table 1:** Demographic characteristics of PW with HBeAg-negative chronic ASCs.

Clinical characteristics	HC	PW	PW-ASCs	Range of reference
Numbers	13	15	21	
Age (years)	26.0. ± 2.98	28.47 ± 3.25	31.9 ± 3.73	
Pregnancy (weeks)	0	26.33 ± 3.11	25.52 ± 3.44	
17-β-estradiol (E2) (pg/mL)	67.91 ± 67.32	4945.26 ± 212.01	>5000	
Progesterone (P4) (ng/mL)	2.74 ± 6.73	89.98 ± 49.99	77.76 ± 40.26	0.00-0.20
HBsAg (IU/mL)	0	0	1052.03 ± 813.53	≥0.05
HBeAg (S/CO)	0.40 ± 0.04	0.02 ± 0.02	0.02 ± 0.03	≥1.00
anti-HBc (S/CO)	0.16 ± 0.31	0.70 ± 0.16	393.19 ± 219.15***	≥1.00
anti-HBe (S/CO)	1.29 ± 0.70	10.89 ± 4.81	96.47 ± 10.99***	<1.00
anti-HBs (IU/L)	236.25 ± 380.59	95.33 ± 272.72^##^	0.51 ± 0.30***	≥10.00
ALT (U/L)	11.85 ± 2.91	15.67 ± 6.66	15.43 ± 8.45	9 - 50
HBV-DNA (IU/mL)	<30	<30	<30	LOD=30

HC, healthy controls; PW, healthy PW; ASCs, chronic asymptomatic HBV carriers; PW-ASCs, PW with chronic ASCs; HBV, hepatitis B virus; HBsAg, hepatitis B surface antigen; HBeAg, hepatitis Be antigen; anti-HBs, hepatitis B surface antibody; anti-HBc, hepatitis B core antibody; anti-HBe, hepatitis Be antibody; E2, 17-β-estradiol; P4, progesterone; ALT, alanine aminotransferase; When E2 level exceeds 50000 pg/mL, this level is considered to be 5000 pg/mL at the time of statistical analysis; LOD: limitation of dection. ^##^PW vs. HC; ***PW-ASCs vs. PW or HC; ^##^P< 0.01, ***P< 0.001.

### 2.2 Serological HBV markers and liver function

Serum levels of HBV markers, including HBsAg, HBsAb (anti-HBs), HBeAg, anti-HBe, and anti-HBc, were tested using a chemiluminescent microparticle immunoassay (Abbott Alinity i, USA). HBV-DNA copies in the serum of the individuals were detected by ABI 7500 fluorescent quantitative PCR (Applied Biosystems, USA). Additionally, serum ALT levels were tested by an automated biochemical analyser (Roche Cobas p 671, Switzerland).

### 2.3 Human peripheral blood mononuclear cells

Fresh venous blood was drawn from the individuals, and PBMCs were separated immediately using density gradient centrifugation with a Ficoll-Hypaque solution (CL5020, Netherlands) based on the manufacturer’s protocol. The human PBMCs were washed at least twice with sterile phosphate-buffered saline (PBS), and then they were resuspended in 200 μL sterile PBS and transferred into sterile tubes for subsequent experiments.

### 2.4 Multicolour flow cytometry analysis

Human PBMCs resuspended in sterile PBS were incubated for 20 min at room temperature with the following fluorochrome-conjugated anti-human antibodies for immunofluorescence staining: anti-human AmCyan-CD3, UV 395-CD4, Percp/Cy5.5-CCR4, PE/Cy7-CXCR5, Brilliant violet (BV) 786-CD25, PE-Texas Red-CD127, PE-CCR6, APC-CCR10, and BV 711-CXCR3 (BD, USA). Matched isotype controls were also used in this study based on the manufacturers’ protocol. The immunophenotyping of CD4^+^ T cells in human PBMCs was performed by flow cytometric analysis with BD LSRFortessa, and the data were analysed using FlowJo™ software v10 (FlowJo, USA).

### 2.5 Serological analysis of cytokines

The levels of interferon-gamma (IFN-γ), interleukin-2 (IL-2), IL-4, IL-6, IL-10, IL-17A and tumour necrosis factor-alpha (TNF-α) were detected according to the manufacturer’s protocol for the human Th1/Th2/Th17 Cytokines Kit (CellGene, China) based on flow cytometric analysis. Briefly, immune microbeads (25 µL) and fluorescence detection reagent (25 µL) were added to serum samples (25 µL), and the mixture was incubated in the dark for 2.5 hours at room temperature. Then, the mixture was washed with PBS and resuspended in PBS buffer (100 µL) for subsequent detection. The analysis of serum cytokines was performed by flow cytometry with NovoCyte D2040R (Agilent Technologies, China).

### 2.6 Statistical analysis

Data were analysed by the Mann−Whitney U test or one-way ANOVA on GraphPad Prism 7 software (GraphPad Software, Inc., CA). Analysis between two unpaired groups was performed by Student’s *t* test or Mann–Whitney U test, and significant differences were confirmed using paired t tests between two paired groups. The correlation analysis was performed using Spearman’s correlation coefficients for the variables between two groups. All *P* values < 0.05 were considered to be significant.

## 3 Results

### 3.1 Demographics of PW with HBeAg-negative chronic ASCs

In this study, 21 PW were chronic ASCs with high serum levels of HBsAg, anti-HBc and anti-HBe but low levels of anti-HBs compared with those of HC and healthy PW. The mean age and serum levels of HBeAg and ALT were not different among the three groups, and the serum titres of HBV-DNA were not undetectable. However, the serum levels of anti-HBs were significantly different between HC and healthy PW (U=29.50, *P*=0.0019) (**Table 1**).

### 3.2 Circulating Tfh cell subsets in PW with HBeAg-negative chronic ASCs

**Figure 1:**
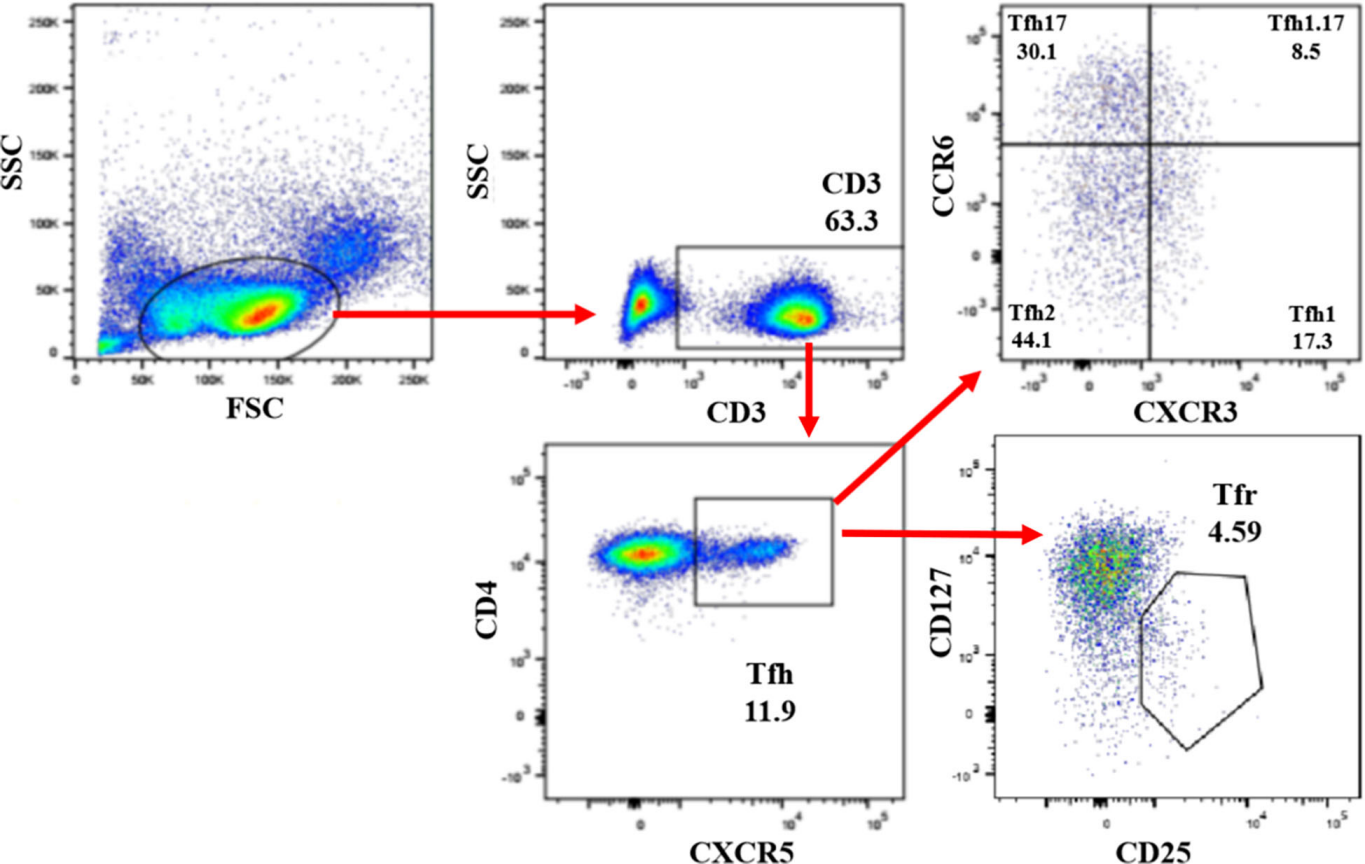
Detection of circulating Tfh cell subsets in PW with HBeAg-negative chronic ASCs. Human PBMCs from HC, healthy PW, and PW with HBeAg-negative chronic ASCs were stained with Fixable Viability Stain 700 (live/dead cells), anti-human CD3, anti-human CD4, anti-human CXCR5, anti-human CXCR3, anti-human CCR6, anti-human CD25, and anti-human CD127 antibodies, and circulating Tfh cell subsets were analysed by flow cytometry. Circulating Tfh cells: CD3^+^CD4^+^CXCR5^+^ Th cells; Tfh1 cells: CXCR3^+^CCR6^-^ Tfh cells; Tfh2 cells: CXCR3^-^CCR6^-^ Tfh cells; Tfh17 cells: CXCR3^-^CCR6^+^ Tfh cells; Tfh1.17 cells: CXCR3^+^CCR6^+^ Tfh cells; Tfr cells: CD25^+^CD127^-^ Tfh cells. PW, pregnant women; ASCs, asymptomatic hepatitis B virus carriers; HC, healthy controls; PBMCs, peripheral blood mononuclear cells; CXCR5, C-X-C motif chemokine receptor type 5; Tfh, T follicular helper cells; Treg, T regulatory cells; CXCR3, C-X-C motif chemokine receptor 3; CCR6, C-C motif chemokine receptor 6.

**Figure 2:**
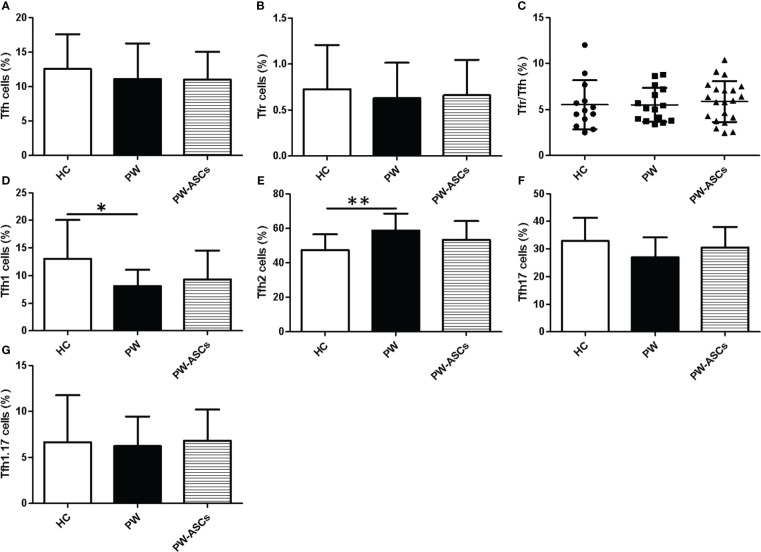
Frequencies of circulating Tfh cell subsets in PW with HBeAg-negative chronic ASCs. **(A)** The proportions of Tfh cells. **(B)** The frequencies of Tfr cells. **(C)** The ratio of Tfr/Tfh cells. **(D)** The frequencies of Tfh1 cells. **(E)** The frequencies of Tfh2 cells. **(F)** The frequencies of Tfh17 cells. **(G)** The frequencies of Tfh1/17 cells. HC: healthy controls; PW, pregnant women; PW-ASCs, PW with chronic ASCs; Tfh, T follicular helper cell; Tfh1, Tfh type-1 cell; Tfh2, Tfh type-2 cell; Tfh17, Tfh type-17 cell; Tfh1.17, Tfh1 and Tfh17 cell; Tfr, follicular regulatory T cell. Data represent the mean ± SD. ^∗^P < 0.05; ** P < 0.05.

Previous studies showed that Tfh (CXCR5^+^CD4^+^CD3^+^) cell subsets played a critical role in viral infections, including chronic HBV infection, which included Tfh type-1 (Tfh1) (CXCR3^+^CCR6^-^), Tfh2 (CXCR3^-^CCR6^-^), Tfh17 (CXCR3^-^CCR6^+^) and Tfh1/17 (CXCR3^+^CCR6^+^) cells based on the expression of CXCR3 and CCR6 (Schmitt et al., 2014; Tuzlak et al., 2021; Yoshitomi and Ueno, 2021; Chen et al., 2022). In this study, circulating CXCR5^+^CD4^+^CD3^+^ Tfh cells in PBMCs from HC, healthy PW, and PW with chronic ASCs were detected by flow cytometry (**Figure 1**). The frequencies of circulating Tfh cells, Tfh17 cells, and Tfh1/17 cells were not significantly changed among the three groups (**Figure 2A, F, G**). Moreover, the frequencies of circulating Tfr (CD127^-^CD25^+^CXCR5^+^CD4^+^CD3^+^) cells and the Tfr/Tfh ratio were also not remarkably varied among the three groups (**Figures 2B, C**). However, the frequencies of circulating Tfh1 cell frequencies were notably decreased; in contrast, Tfh2 cells were significantly increased in PW compared to HC (**Figures 2D, E**). Interestingly, the frequencies of circulating Tfh2 cells were positively associated with P4 levels, but the frequencies of circulating Tfh17 cells were negatively associated with P4 levels in healthy PW (**Table 2**), but not in PW with chronic ASCs (data not shown). Additionally, there was no significant correlation between circulating Tfh cell subsets and serum HBV markers, ALT levels or E2 levels in HC, PW, and PW with chronic ASCs (data not shown).

**Table 2:** The correlation of CD4^+^ Th cells and P4 levels in healthy PW.

CD4^+^ Th cells	Spearman r	*P* value	CD4^+^ Th cells	Spearman r	*P* value
Th1/P4	0.0500	0.8595	Tfh/P4	-0.2750	0.3212
**Th2/P4**	**0.5357**	0.0396*	Tfh1/P4	-0.2878	0.2983
**Th17/P4**	**-0.6964**	0.0039**	**Tfh2/P4**	**0.5308**	0.0418*
Th22/P4	-0.4615	0.0833	**Tfh17/P4**	**-0.6964**	0.0039**
Th1.17/P4	-0.5107	0.0517	Tfh1.17/P4	-0.4039	0.1354
Treg/P4	0.1162	0.6801	Tfr/P4	0.2964	0.2834

PW, pregnant women; P4, progesterone; Treg, T regulatory cell; Tfh, T follicular helper cell; Tfh1, Tfh type-1 cell; Tfh2, Tfh type-2 cell; Tfh17, Tfh type-17 cell; Tfh1.17, Tfh1 and Tfh17 cell; Tfr, follicular regulatory T cell; *P < 0.05, **P < 0.01. Bold values indicates statistically significant results.

### 3.3 Circulating non-Tfh cell subsets in PW with HBeAg-negative chronic ASCs

**Figure 3:**
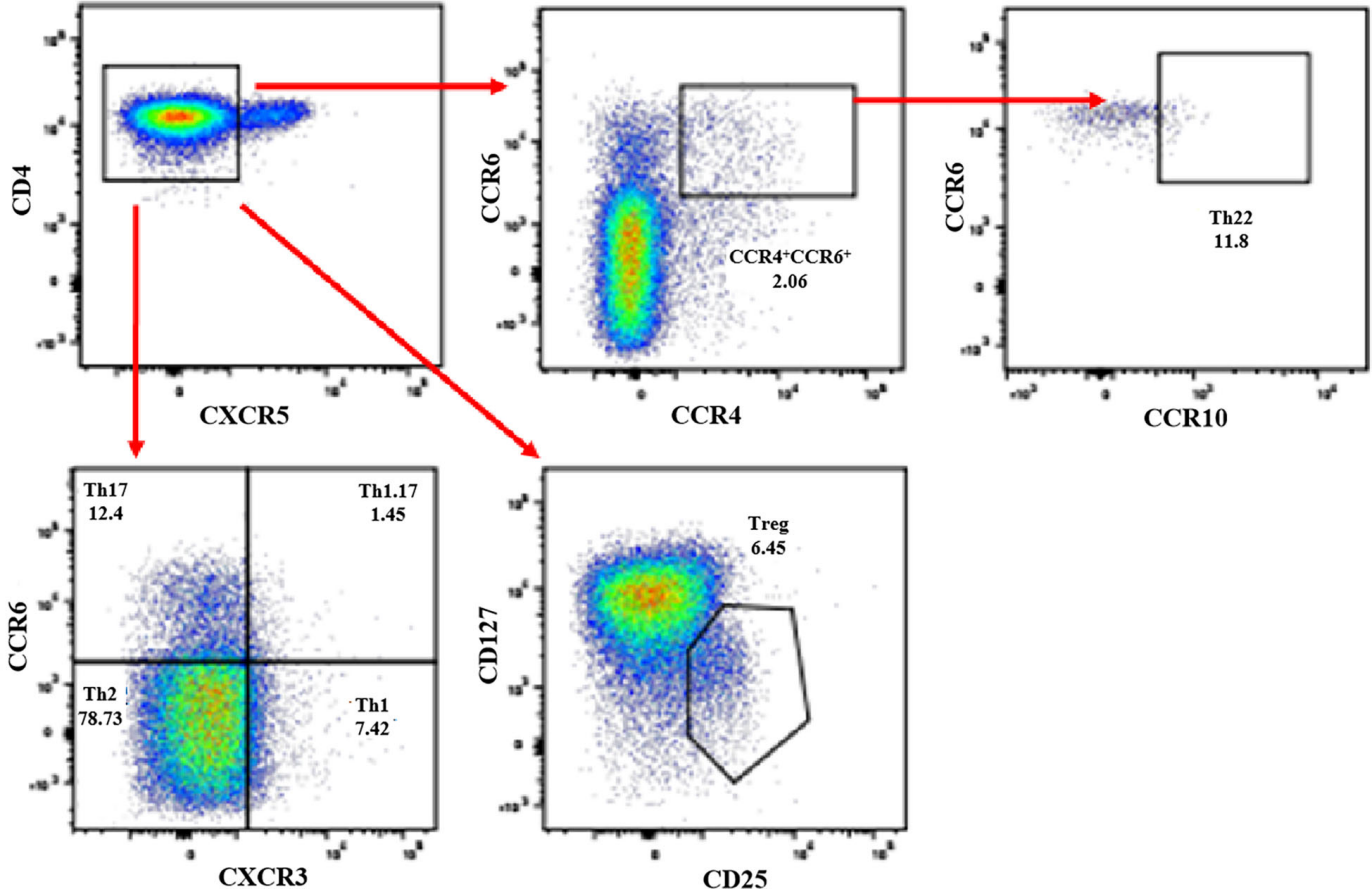
Detection of non-Th cell subsets in PW with HBeAg-negative chronic ASCs. Human PBMCs from HC, PW, and PW with HBeAg-negative chronic ASCs were stained with anti-human CD3, anti-human CD4, anti-human CXCR5, anti-human CXCR3, anti-human CCR6, anti-human CD25, and anti-human CD127 antibodies, and non-Tfh cell subsets were analysed by flow cytometry. Th1 cells: CD3^+^CD4^+^CXCR5^-^CXCR3^+^CCR6^-^ Th cells; Th2 cells: CD3^+^CD4^+^CXCR5^-^CXCR3^-^CCR6^-^ Th cells; Th17 cells: CD3^+^CD4^+^CXCR5^-^CXCR3^-^CCR6^+^ Th cells; Th1.17 cells: CD3^+^CD4^+^CXCR5^-^CXCR3^+^CCR6^+^ Th cells; Th22 cells: CD3^+^CD4^+^CXCR5^-^CCR4^+^CCR6^+^CCR10^+^ Th cells; Treg cells: CD3^+^CD4^+^CXCR5^-^CD25^+^CD127^-^ Th cells. PW, pregnant women; ASCs, asymptomatic hepatitis B virus carriers; HC, healthy controls; PBMCs, peripheral blood mononuclear cells; CXCR5, C-X-C motif chemokine receptor type 5; Treg, T regulatory cells; CXCR3, C-X-C motif chemokine receptor 3; CCR6, C-C motif chemokine receptor 6.

**Figure 4:**
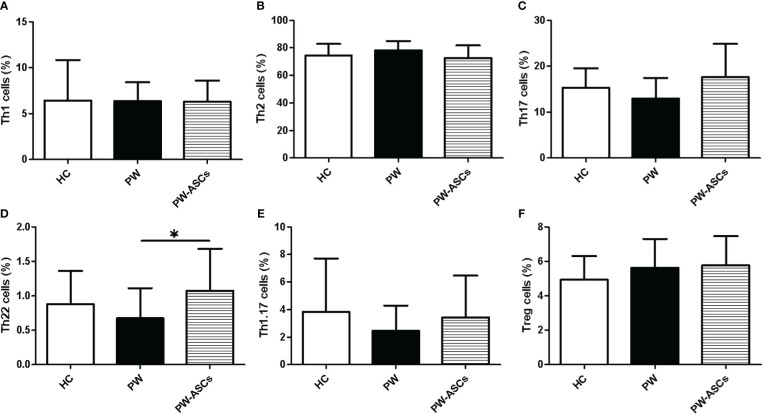
Frequencies of circulating non-Tfh cell subsets in PW with HBeAg-negative chronic ASCs. **(A)** The proportions of Th1 cells. **(B)** The frequencies of Th2 cells. **(C)** The frequencies of Th17 cells. **(D)** The frequencies of Th1.17 cells. **(E)** The frequencies of Th22 cells. **(F)** The frequencies of Treg cells. HC, healthy controls; PW, pregnant women; PW-ASCs, PW with chronic ASCs. Data represent the mean ± SD. ^∗^P < 0.05.

Previous reports indicated that non-Tfh cells, including Th1 (CD3^+^CD4^+^CXCR5^-^CXCR3^+^CCR6^-^), Th2 (CD3^+^CD4^+^CXCR5^-^CXCR3^-^CCR6^-^), Th17 (CD3^+^CD4^+^CXCR5^-^CXCR3^-^CCR6^+^), Th22 (CD3^+^CD4^+^CXCR5^-^CCR4^+^CCR6^+^CCR10^+^), and Treg (CD3^+^CD4^+^CXCR5^-^CD25^+^CD127^-^) cells, played an important role in the pathogenesis of PW or chronic HBV infection (Shrivastava et al., 2013; Zhao et al., 2014; Liang et al., 2020; Huang et al., 2021; Sirilert and Tongsong, 2021; Islam et al., 2022; Zhang et al., 2022). In this study, the frequencies of non-Tfh cell subsets in PW with HBeAg-negative chronic ASCs were analysed by gating CD3^+^CD4^+^ T cells from human PBMCs using flow cytometry (**Figure 3**). The results showed that the frequencies of circulating Th22 cells were only notably increased in PW with chronic ASCs in comparison with PW but were not obviously different between HC and PW or PW with chronic ASCs (**Figure 4D**). Moreover, the frequencies of other non-Tfh cells, including Th1/2/17, Th1/17 and Treg cells, were not significantly changed among HC, PW and PW with chronic ASCs (**Figures 4A–C, E, F**). Interestingly, the frequencies of circulating Th2 cells were positively associated with P4 levels, but the frequencies of circulating Th17 cells were negatively associated with P4 levels in healthy PW (**Table 2**), but not in PW with chronic ASCs (data not shown). Additionally, a significant correlation was not observed between the frequencies of circulating non-Tfh cell subsets and serum HBV markers, ALT levels or E2 levels in HC, PW, and PW with chronic ASCs (data not shown).

### 3.4 Serum levels of cytokines in PW with HBeAg-negative chronic ASCs

**Figure 5:**
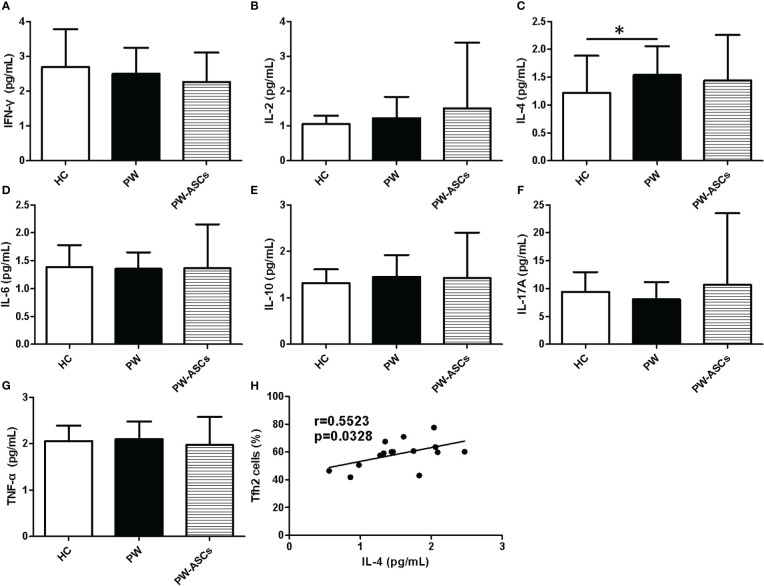
Expression levels of seven cytokines in serum from PW with HBeAg-negative chronic ASCs. **(A)** The levels of IFN-γ. **(B)** The levels of IL-2. **(C)** The levels of IL-4. **(D)** The levels of IL-6. **(E)** The levels of IL-10. **(F)** The levels of IL-17A. **(G)** The levels of TNF-α. **(H)** The association of Tfh2 cell frequencies and serum IL-4 levels in healthy PW. PW, pregnant women; ASCs, asymptomatic hepatitis B virus carriers; HBeAg, hepatitis Be antigen; IFN-γ, interferon-gamma; IL-2, interleukin-2; IL-4, interleukin-4; IL-6: interleukin-6; IL-10, interleukin-10; IL-17A, interleukin-10A; TNF-α, tumor necrosis factor-α; Tfh, T follicular helper cell; Tfh2, Tfh type-2 cell. Data represent mean ± SD, *P < 0.05.

Serum cytokines play a critical role in maintaining a successful pregnancy, such as IL-4, IL-10, and IFN-γ (Huang et al., 2021; Sirilert and Tongsong, 2021). To further explore the immune status of PW with chronic ASCs, the levels of seven key cytokines (IFN-γ, IL-2, IL-4, IL-6, IL-10, IL-17A, TNF-α) were detected in serum from HC, PW and PW with chronic ASCs by flow cytometry. Interestingly, serum levels of IL-4 were significantly elevated in PW compared to HC and were also positively related to the frequencies of Tfh2 cells in PW (**Figures 5C, H**) (**Table 3**). Additionally, a decreasing trend in serum IFN-γ levels was observed in HC, PW and PW with chronic ASCs but was not notably different among the three groups (**Figure 5A**). Moreover, serum levels of IL-2, IL-6, IL-10, IL-17A, and TNF-α were not notably different among the three groups, and a significant correlation between these cytokine levels and the frequencies of non-Tfh cell subsets was not observed among the three groups (**Figures 5B, D–G**; **Table 4**).

**Table 3:** Correlation of CD4^+^ Th cells and serum cytokines in healthy PW.

CD4^+^ Th cells	Spearman r	*P* value	CD4^+^ Th cells	Spearman r	*P* value
Th1/IFN-γ	-0.1250	0.6571	Tfh1/IFN-γ	0.1662	0.5538
Th2/IL-4	0.5000	0.0577	**Tfh2/IL-4**	**0.5523**	0.0328*
Th17/IL-17A	-0.2179	0.4354	Tfh17/IL-17A	-0.08929	0.7517
Th22/IL-6	-0.06082	0.8295	Tfh/IL-6	0.05357	0.8496
Th22/TNF-α	-0.4920	0.0625	Tfr/IL-10	0.2179	0.4354
Treg/IL-10	-0.1930	0.4907	Tfr/IL-2	0.02326	0.9344
Treg/IL-2	-0.08863	0.7534			

PW, pregnant women; IFN-γ, interferon-gamma; IL-2, interleukin-2; IL-4, interleukin-4; IL-6, interleukin-6; IL-10, interleukin-10; IL-17A, interleukin-17A; TNF-α, tumor necrosis factor-α; Treg, T regulatory cell; Tfh, T follicular helper cell; Tfh1, Tfh type-1 cell; Tfh2, Tfh type-2 cell; Tfh17, Tfh type-17 cell; Tfh1.17, Tfh1 and Tfh17 cell; Tfr, follicular regulatory T cell; *P < 0.05.

**Table 4:** Correlation of CD4^+^ Th cells and serum cytokines in PW with HBeAg-negative chronic ASCs.

CD4^+^ Th cells	Spearman r	*P* value	CD4^+^ Th cells	Spearman r	*P* value
Th1/IFN-γ	0.01754	0.9398	Tfh1/IFN-γ	-0.3156	0.1635
Th2/IL-4	-0.1299	0.5746	Tfh2/IL-4	0.07210	0.7561
Th17/IL-17A	0.1156	0.6178	Tfh17/IL-17A	0.5714	0.8057
Th22/IL-6	-0.03899	0.8668	Tfh/IL-6	-0.003897	0.9866
Th22/TNF-α	0.04029	0.8624	Tfr/IL-10	-0.06693	0.7732
Treg/IL-10	0.2164	0.3462	Tfr/IL-2	-0.003899	0.9866
Treg/IL-2	0.1475	0.5234			

PW, pregnant women; ASCs, asymptomatic hepatitis B virus carriers; HBeAg, hepatitis Be antigen; IFN-γ, interferon-gamma; IL-2, interleukin-2; IL-4, interleukin-4; IL-6, interleukin-6; IL-10, interleukin-10; IL-17A, interleukin-17A; TNF-α, tumor necrosis factor-α; Treg, T regulatory cell; Tfh, T follicular helper cell; Tfh1, Tfh type-1 cell; Tfh2, Tfh type-2 cell; Tfh17, Tfh type-17 cell; Tfh1.17, Tfh1 and Tfh17 cell; Tfr, follicular regulatory T cell; * P< 0.05.

## 4 Discussion

In the present study, we systematically assessed the frequencies of human circulating CD4^+^ Th cell subsets in PBMCs from healthy controls (HC), pregnant women (PW), and PW with HBeAg-negative chronic ASCs. The results showed that HBV-DNA and ALT levels were not different among HC, healthy PW, and PW with HBeAg-negative chronic ASCs. Moreover, the frequencies of circulating Tfh2 cells were surprisingly increased in PW compared to HC and were also positively associated with evaluated serum IL-4 levels in PW. However, Tfh1 cell frequencies were significantly reduced in PW compared with HC. Interestingly, serum P4 levels were positively associated with the frequencies of circulating Tfh2 or Th2 cells but were negatively related to the frequencies of circulating Tfh17 or Th17 cells in healthy PW. Additionally, Th22 cell frequencies were also remarkably expanded in PW with chronic ASCs compared to PW, but the frequencies of other CD4^+^ Th cell subsets and the serum levels of some key cytokines were not significantly changed among HC, PW, and PW with HBeAg-negative chronic ASCs. In summary, these data suggest a critical role of CD4^+^ Th cell subsets during pregnancy, which may help to provide potential evidence for PW withHBeAg-negative chronic ASCs and do not receive antenatal antiviral prophylaxis.

Chronic asymptomatic HBV carriers (ASCs) with HBeAg-negative are predominant among chronic ASCs, including women of childbearing age, and can also develop a potential risk of high morbidity due to HBV-related fatal liver diseases, mostly hepatocellular carcinoma and cirrhosis (Kumar et al., 2009; Organization, 2016; Cui et al., 2022; Lian et al., 2022; Wang and Chen, 2022). Previous reports indicated that the levels of serum HBV-DNA, viral antigens (HBsAg, HBeAg) and ALT were mostly normal or stable in chronic ASCs with/without HBeAg-negative status, including pregnancy (Cheung et al., 2021; Hsu et al., 2022; Wang et al., 2022). Our results showed that low HBV-DNA and ALT levels were not significantly different among HC, PW, and PW with HBeAg-negative ASCs. Accumulating evidence has not shown that HBV antiviral therapy obviously affects clinical or serological outcomes during pregnancy (Terrault et al., 2021). Although prenatal antiviral prophylaxis for chronic ASCs with and without HBeAg who have low viral titres is not commonly performed during pregnancy, regular follow-up is necessary to identify maternal or foetal indications for timely antiviral therapy (Cui et al., 2016; Funk et al., 2021).

It is well known that the foetus is semiallogenic to the maternal host during pregnancy, and maternal-foetal immune tolerance plays a key role in ensuring a successful pregnancy (Piccinni et al., 2021; Moldenhauer et al., 2022). Disturbance of the maternal immune system is an important factor that leads to foetal abortion, which mainly involves immune cells and cytokines, such as Tfh, Th1/2/17 and Treg cells, in the peripheral blood of pregnant women (Wang et al., 2020; Monteiro et al., 2021; Piccinni et al., 2021). Previous reports indicated that expanded circulating Tfh cells contributed to humoral immunity during pregnancy and were able to secrete large amounts of IL-6, IL-21 and IL-10 by helping B cells (Monteiro et al., 2017; Wang et al., 2020; Monteiro et al., 2021). Based on CXCR3 and CCR6 expression, circulating Tfh cells are divided into four subsets: Tfh1 (CXCR3^+^CCR6^-^), Tfh2 (CXCR3^-^CCR6^-^), Tfh17 (CXCR3^-^CCR6^+^), and Tfh1/17 (CXCR3^+^CCR6^+^) (Maecker et al., 2012; Huang et al., 2019; Liu et al., 2021; Feng et al., 2022). Circulating Tfh2 and Tfh17 cells can effectively help naïve B cells differentiate into plasma cells to generate antibodies, including neutralizing antibodies against immunization or pathogens such as HBV and human immunodeficiency virus (HIV), and autoantibodies to mediate the development of autoimmune diseases, including systemic lupus erythematosus (SLE) and rheumatoid arthritis (RA), while Tfh1 cells lose the ability to assist naïve B cells but can contribute to the differentiation of memory B cells (Kubo et al., 2017; Vella et al., 2017; Wang et al., 2018; Lin et al., 2019; Cui et al., 2021). Our data showed that the frequencies of circulating Tfh cells, Tfh17 cells, Tfh1/17 cells and Tfr cells were not significantly changed among HC, PW, and PW with ASCs, but the frequencies of circulating Tfh2 cells were significantly increased and were positively associated with elevated levels of serum IL-4. Tfh1 cell frequencies were notably decreased in healthy PW compared to HC. These findings suggest that increased Tfh2 cells and decreased Tfh1 cells might improve pregnancy success by supporting humoral immunity, and HBV infection also affects Tfh cell subsets and functional characteristics. However, Monteiro C et al. reported that no significant difference was found in the frequencies of circulating IFN-γ^+^ Tfh1, IL-4^+^ Tfh2, and IL-17^+^ Tfh17 cells stimulated with phorbol 12-myristate 13-acetate (PMA) and ionomycin between PW and nPW (Monteiro et al., 2017; Wang et al., 2020). These findings may be relevant to the definition of different Tfh cell subtypes or activated status. Additionally, a previous report showed that lower frequencies of splenic CD4^+^CXCR5^+^Bcl-6^+^ Tfh cells could contribute to successful pregnancies in mice (Fröhlich et al., 2020). The phenotypes and function of Tfh cell subsets during human and mouse pregnancy need to be further elucidated to contribute to successful pregnancy.

Accumulating evidence indicates that hormone levels including E2 and P4, favour the expansion of CD4^+^ Th cell subsets, including Treg, Th2, and Tfh cells, and inhibit the number of Th17 and Th1 cells during human and mouse pregnancy (Monteiro et al., 2017; Fröhlich et al., 2020; Wang et al., 2020; Monteiro et al., 2021). Interestingly, our results showed a positive association between serum P4 levels and frequencies of Tfh2 or Th2 cells and a negative relation between serum P4 levels and the frequencies of Tfh17 or Th17 cells in healthy PW, but not in PW with chronic ASCs, which implied that HBV infection affected the hormone levels of PW. Unfortunately, E2 levels were too high to measure in our study, which affected further analysis of some of the results, which will be explored in the future study. These findings further indicated that hormone levels in healthy PW or PW with chronic HBV infection affected the changes in CD4^+^ Th cell subsets.

The immune tolerance of the maternal-foetal microenvironment is essential for a successful pregnancy. The cumulative evidence suggests that Treg cells and Th2 cells promote allograft tolerance by repressing Th1 and Th17 cells that are accountable for spontaneous abortion, which can secrete IL-10, TGF-β and IL-4 cytokines, respectively (Liang et al., 2020; Wang et al., 2020). However, increased Th17 and Th22 cells can maintain a successful pregnancy by protecting trophoblasts from pathogens and inflammatory responses caused by intrauterine infection, including various viruses and bacteria (Logiodice et al., 2019; Wang et al., 2020; Piccinni et al., 2021). Our data showed that the population of Th22 cells was only significantly expanded in PW with chronic ASCs in comparison with PW, which implied that Th22 cells may play an important role in resisting HBV infection in PW with chronic ASCs. Th22 cells typically produce IL-22 deprived of IL-4, IFN-γ, and IL-17, which are also defined as CD3^+^CD4^+^CXCR5^-^CCR4^+^CCR6^+^CCR10^+^ CD4^+^ Th22 cells, and Th22 cells are usually elevated when the human body is infected by viruses, including HBV, and play a critical role in resisting the virus and controlling inflammation (Maecker et al., 2012; Logiodice et al., 2019; Jiang et al., 2021). Moreover, the interplay of IL-22/IL-22 receptor 1 (IL-22R1) contributes to the promotion of trophoblast survival and pregnancy maintenance (Wang et al., 2020). However, the frequencies of other CD4^+^ Th cell subsets, including Th1, Th2, Th17, and Treg cells, and the serum levels of cytokines, such as IFN-γ, IL-2, IL-17, TNF-α, and IL-10, were not significantly changed among HC, healthy PW, and PW with ASCs in this study. Although these results with no statistical deviation may be partially related to some factors, including the number of enrolled subjects and the period of pregnancy, these findings provided potential evidence for why PW with chronic ASCs did not receive antenatal antiviral prophylaxis based on the immune status of the pregnant women. Previous reports indicated that CD4^+^ Th cell subpopulations had marked plasticity, had a certain conversion relationship with each other and could secrete the corresponding cytokines (Logiodice et al., 2019; Wan et al., 2020; Jiang et al., 2021; Tuzlak et al., 2021; Feng et al., 2022; Feng et al., 2022). These findings suggest that disorders of CD4^+^ T-cell subsets can easily result in foetal abortion, and their relative balance is critical to successful pregnancy.

## 5 Conclusion

Reproduction is one key factor for the survival and development of humans, and successful gestation is responsible for human reproduction. Parallel immune homeostasis is indispensable for protecting the semiallogeneic foetus from harmful maternal immune microenvironments during pregnancy (Piccinni et al., 2021). Pregnant women with HBeAg-negative chronic ASCs are one of the most important populations among women with chronic ASCs, and there is some debate about who needs antenatal antiviral therapy (Cui et al., 2016; Organization WH, 2020; Funk et al., 2021; Terrault et al., 2021). Our results showed expanded circulating Tfh2 cells and associated serum IL-4 levels, as well as decreased Tfh1 cells, in PW. Moreover, serum P4 levels expanded the frequencies of Tfh2 or Th2 cells but reduced frequencies of Tfh17 or Th17 cells in healthy PW, which promoted successful pregnancy. Additionally, increased Th22 cells in PW with HBeAg-negative chronic ASCs maybe contribute to the promotion of trophoblast survival and pregnancy maintenance. Taken together, these findings indicated that parallel immune homeostasis is critical for successful pregnancy, which could partially explain why PW with chronic ASCs did not receive antenatal antiviral prophylaxis.

## Data availability statement

The original contributions presented in the study are included in the article/supplementary material. Further inquiries can be directed to the corresponding authors.

## Ethics statement

The studies involving human participants were reviewed and approved by This study was approved by the Medical Ethical Committee of the Women’s Hospital, Zhejiang University School of Medicine. The patients/participants provided their written informed consent to participate in this study. Written informed consent was obtained from the individual(s) for the publication of any potentially identifiable images or data included in this article.

## Author contributions

GF and DC wrote the manuscript and performed the experiment. GF and YS collected the clinical data and samples. GF, YL, CY and DC analyzed the data. DC, YS, SW and YZ revised the manuscript. DC, YZ and YoZ conceived the topic and revised the manuscript. All authors contributed to the article and approved the submitted version.
